# Mitigating gender bias in student evaluations of teaching

**DOI:** 10.1371/journal.pone.0216241

**Published:** 2019-05-15

**Authors:** David A. M. Peterson, Lori A. Biederman, David Andersen, Tessa M. Ditonto, Kevin Roe

**Affiliations:** 1 Department of Political Science, Iowa State University, Ames, Iowa, United States of America; 2 Department of Ecology, Evolution, and Organismal Biology, Iowa State University, Ames, Iowa, United States of America; 3 Department of Natural Resource Ecology and Management, Iowa State University, Ames, Iowa, United States of America; Rice University, UNITED STATES

## Abstract

Student evaluations of teaching are widely believed to contain gender bias. In this study, we conduct a randomized experiment with the student evaluations of teaching in four classes with large enrollments, two taught by male instructors and two taught by female instructors. In each of the courses, students were randomly assigned to either receive the standard evaluation instrument or the same instrument with language intended to reduce gender bias. Students in the anti-bias language condition had significantly higher rankings of female instructors than students in the standard treatment. There were no differences between treatment groups for male instructors. These results indicate that a relatively simple intervention in language can potentially mitigate gender bias in student evaluation of teaching.

## Introduction

Student evaluations of teaching (SET) are a ubiquitous form of evaluation. At many colleges and universities, student evaluations are the primary data used to evaluate teaching effectiveness and contributes to tenure and promotion packages [1]. However, there is a growing literature in multiple disciplines that documents bias in these evaluations, including gender bias [2–7]. In particular, female instructors tend to be evaluated more critically than their male peers, even when there are no differences in the quality of instruction or when the gender of the instructor is experimentally and randomly assigned [5]. These biases go as far as influencing even objective assessments of instructors (e.g. speed in the return of grades) [5]. Across a range of indicators, the experimental research shows that gender bias is approximately 0.50 points on a five-point scale [5]. The potential for gender biases in SET leads many academics to question their use and to a growing conversation about the need for alternative mechanisms for evaluating instructors [8].

Despite the growth in attempts to document gender biases in SET, there are few effective evidence-based tools for mitigating these biases. We hypothesized that if bias was informing students’ assessments of teaching then an intervention that makes students aware of their potential biases may partially mitigate the effects of this bias, and thus improve the SET scores of female instructors.

## Hypotheses

The belief that SETs contain biases against female instructors is widespread, even if the empirical evidence is decidedly mixed [9, 10]. Both observational and experimental evidence does provide some evidence that suggests students do harbor some gender biases that filter into their SET [2–7, 9, 10]. These negative biases are likely to be implicit, meaning they are automatically activated, unintentional, and occur below the conscious awareness of the individual [11]. The difficulty is that many other confounding effects tend to correlate with the gender of the instructor. For example, female instructors may anticipate the bias and compensate by working harder and being better instructors than their male colleagues.

However, getting people to overcome these biases can be difficult [12]. There is ample social psychology literature suggests several possible interventions to overcome these biases [13]. The most fruitful approaches emphasize the importance of getting subjects to control their stereotypes or motivating the respondents to focus on the accuracy of their judgments [14–16]. Unfortunately, these attempts can often result in rebound effects, where the subject’s stereotypes become even more powerful [17]. The problem is that these biases essentially become a habit and an ingrained part of a person’s thinking, making them more difficult to reduce than explicit expressions of prejudice. Overcoming the influence of implicit biases requires 1) awareness of the implicit biases, 2) the motivation to break the “habit” of their use, and 3) training in ways to overcome the implicit bias [15, 16, 18].

Long-term reductions in the biases of students are beyond a simple intervention in student surveys of instructor effectiveness. It may be possible, however, to limit the immediate use of biases in SET by cuing students to be aware of their biases, providing motivation to not rely on them, and suggesting alternatives to their stereotypes. We hypothesize that students provided with cues that make them aware of gender biases and motivate them to rely on less stereotypical considerations about their instructor will result in more positive ratings of female instructors compared to students who do not receive these cues. We expect the bias cues to not have an effect on male instructors. While there are certainly stereotypes about males, they are less negative in this context. Furthermore, the cue we provide (see below) is directed specifically at biases about female instructors.

The sex of the student may also shape how students respond to efforts to mitigate biases. The literature on gender bias in SET often tests for a difference based on the sex of the student, with mixed findings [4, 19]. Male students may be more likely to harbor biases against female instructors, which may result in stronger treatment effects for male students than for female students.

## Material and methods

To test these hypotheses, we conducted an experiment in four large introductory courses in Spring of 2018: two introduction to biology courses and two introduction to American politics courses. Replication data are available at the lead author’s Dataverse page (https://doi.org/10.7910/DVN/AB4ZAV). The experiment received approval from the Institutional Review Board at Iowa State University (#18–183). The Board ruled that the study was exempt from full review because it was conducted in an established or commonly accepted educational setting. Additionally, the research did not need to acquire informed consent and did not require parental consent if any of the students were under 18. Within each pair of courses, one section was taught by a female instructor and one section was taught by a male instructor. All four instructors are White. At this university, SETs are conducted online and students receive an email with the link to the evaluation of their instructor for each course. For each course, we randomized the students into one of two conditions. In the control condition, students received the standard SET survey for their department. In the treatment condition, the solicitation and the evaluation instrument used language that we expected to mitigate gender biases. The added language was:

“Student evaluations of teaching play an important role in the review of faculty. Your opinions influence the review of instructors that takes place every year. Iowa State University recognizes that student evaluations of teaching are often influenced by students’ **unconscious** and **unintentional** biases about the race and gender of the instructor. Women and instructors of color are systematically rated lower in their teaching evaluations than white men, even when there are no actual differences in the instruction or in what students have learned.

As you fill out the course evaluation please keep this in mind and make an effort to resist stereotypes about professors. Focus on your opinions about the content of the course (the assignments, the textbook, the in-class material) and not unrelated matters (the instructor’s appearance).”

Because we randomized within the courses instead of across the courses, we can directly compare the SETs across the conditions without concern that there were other confounding variables about the instructors or the course material that would be responsible for any differences.

We designed the prompt to overcome the influence of bias (steps 1–3 above). Specifically, the prompt makes the student aware of the possibility of bias (step 1), attempts to motivate them to suppress its effects (step 2), and provides cues about what other considerations he or she could use when answering the questions about their instructor (step 3).

The dependent variables in these analyses are three questions that evaluate the course and the instructor. Each student was asked: “Your overall rating of this instructor is?” “What is your overall rating of the instructor’s teaching effectiveness?” “And Your overall rating of this course is?” These questions are the default ones used in the two departments. We refer to these questions as, respectively, Instructor, Effectiveness, and Course. Each of the questions has a five-point scale, with higher values representing more positive evaluations. Given the ordered nature of these questions, we will model the responses by ordered logits.

Because we hypothesize that the effect of the treatment cue will depend on the sex of the instructor, we combined the SET for the two female instructors into one model and the SET of the two male instructors into another. Our analyses present these models separately. The evaluation instrument also included the other standard questions for the University’s SET, including the self-reported gender of the student (see online appendix for full questionnaire) and we use this measure to test the hypotheses that these effects are conditional on the gender of the student. For these analyses, female students are coded as “one” and male students as “zero.”

The survey also included several standard measures about the students and their assessments of other aspects of the course that we will use to test the balance of the conditions. Specifically, the evaluation asked the students their assessment of the textbook, the in-class activities, how much time the student spent on the course, how much time spent they think is valuable, their GPA, the expected grade in the course, and their class in school. (S1 File contains the full script of the course evaluation survey). Given the random assignment to the treatment and the control, we expect no differences in these measures across conditions.

To test the main hypotheses, we used two tests: a t-test and an ordered logit that account for the ordinal nature of the dependent variables. In both cases the results are consistent. For the tests of the student gender hypothesis, we only conducted the ordered logits that both account for the ordinal dependent variables and the inclusion of additional variables. We continue to present the results of the male and female instructors as separate models Finally, to make the effects clear, we include an indicator of the sex of the student and then separate indicators for the treatment effect on male students and the effect of the treatment on female students and we use post-hoc tests of the equality of these coefficients test if the effects are significantly different based on the sex of the student.

## Results

Before testing the hypotheses, we evaluated the balance of the conditions for the other items included in the survey and there was no evidence of imbalance (see S1 Table). The proportion of male students and the mean levels of the students’ class in school, the students’ expected grade in the class, and their GPA were the same between the treatment and the control conditions. Furthermore, if we separate the data based on the gender of the instructor, these results do not change. The randomization appears to be successful.

**Fig 1:**
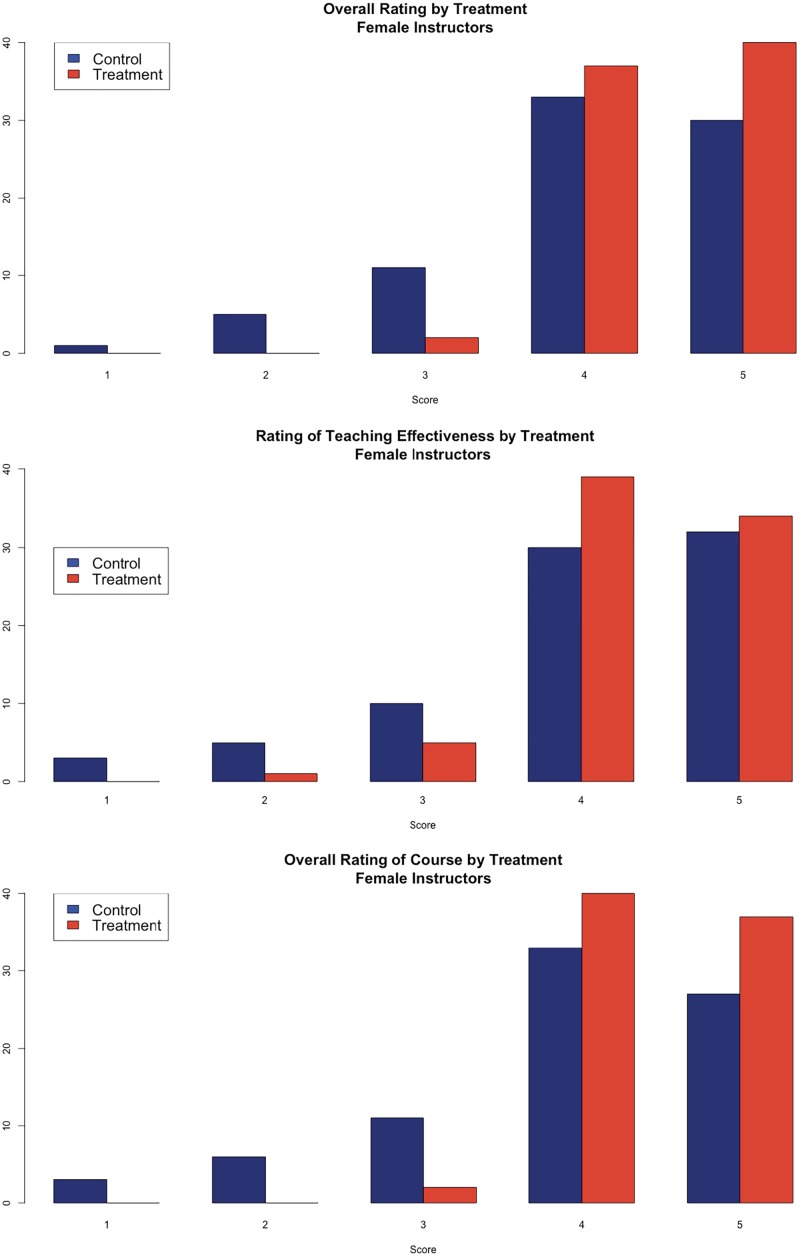
Analysis of student evaluations of teaching by experimental condition (female faculty). ** (A)** Student’s overall rating of the instructor. Higher values are more positive ratings. **(B)** Student’s rating of the instructor’s teaching effectiveness. Higher values are more positive ratings. **(C)** Student’s overall rating of the course. Higher values are more positive ratings. For each panel, the left (blue) bar are the students in the control condition and the right (red) bar are students in the treatment condition.

Fig 1 presents the distribution of the answers to the three main evaluation questions for the female faculty, where the blue bars are from the control and the red bars are from the treatment condition. For each of the evaluation questions, there were significantly higher scores for the female faculty in the treatment condition compared to the control. The answers to the overall evaluation of teaching are 0.41 points higher in the treatment condition (t-value of mean difference = 3.32, p<0.01). The difference in the means for the teaching effectiveness question is 0.30 points (t-value of mean difference = 2.17, p<0.05). For the overall evaluation of the course, the treatment condition is 0.51 points higher than the control (t-value of mean difference = 3.77, p<0.01).

**Fig 2:**
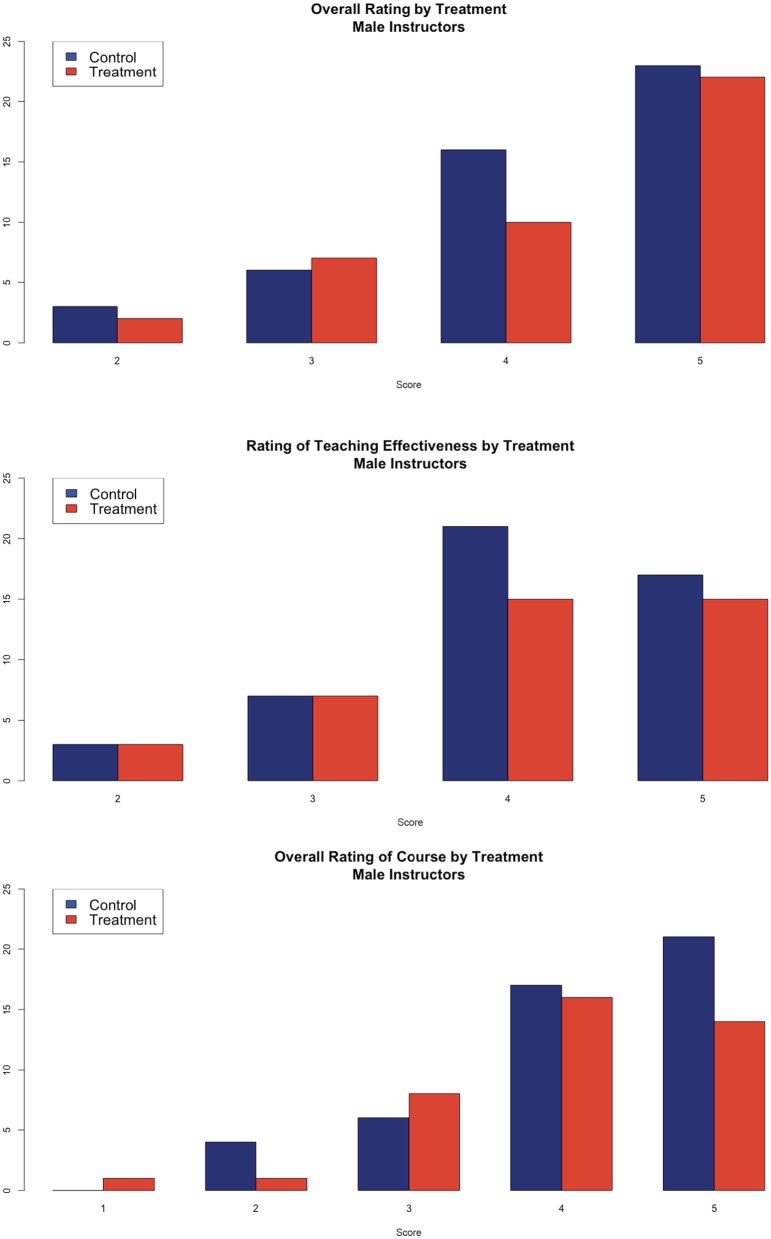
Analysis of student evaluations of teaching by experimental condition (male faculty). **(A)** Student’s overall rating of the instructor. Higher values are more positive ratings. **(B)** Student’s rating of the instructor’s teaching effectiveness. Higher values are more positive ratings. **(C)** Student’s overall rating of the course. Higher values are more positive ratings. For each panel, the left (blue) bar are the students in the control condition and the right (red) bar are students in the treatment condition.

The language seems to have had a positive effect for female faculty and no effect for male faculty (Fig 2). The difference in the overall rating of male faculty was only 0.04 points (t-value of mean difference = 0.20, p>0.10). There are similarly small differences in the teaching effectiveness (difference of 0.03, t-value of mean difference = 0.17, p>0.10) and the overall evaluation of the class (difference of 0.12, t-value of mean difference = 0.60, p>0.10). The ordinal nature of the dependent variables makes the t-tests potentially misleading. Table 1 provides similar models estimated as ordered logits. The results are consistent with the t-tests, summarized above. For female faculty, the treatment has a significant positive effect on the evaluations of the overall rating of the instructor and the overall rating of the course. Unlike the results of the t-tests, there is a positive, but not significant effect of the intervention on the rating of the instructor’s teaching effectiveness. For male faculty, Table 2 shows that there are no significant effects of the intervention for any of the questions.

**Table 1:** Treatment effects on SET for female faculty (ordered logit models).

Variable	Instructor	Effectiveness	Course
Treatment (intervention = 1)	0.82 (0.31)*	0.42 (0.30)	0.92 (0.31)*
Threshold 1	-7.73 (1.01)	-3.76 (0.60)	-3.59 (0.59)
Threshold 2	-2.89 (0.43)	-2.61 (0.37)	-2.43 (0.36)
Threshold 3	-1.63 (0.28)	-1.51 (0.26)	-1.41 (0.26)
Threshold 4	0.68 (0.23)	0.57 (0.23)	0.91 (0.24)
N	159	159	159

* p<0.05

**Table 2:** Treatment effects on SET for male faculty (ordered logit models).

Variable	Instructor	Effectiveness	Course
Treatment (intervention = 1)	0.12 (0.40)	-0.04 (0.40)	-0.29 (0.40)
Threshold 1	-2.77 (0.49)	-2.63 (0.46)	-4.60 (1.02)
Threshold 2	-1.31 (0.32)	-1.24 (0.31)	-2.76 (0.47)
Threshold 3	0.32 (0.28)	0.54 (0.28)	-1.36 (0.32)
Threshold 4	-	-	0.28 (0.28)
N	89	88	88

Note that there are only three thresholds in the first two columns because no respondents used the lowest category of those questions for either of the male faculty.

The second hypothesis is that the effect of the treatment will depend on the sex of the student. Tables 3 and 4 present the results of the ordered models for the female and male instructors and separate out the effects of the intervention based on the self-reported sex of the student. For the models predicting evaluations of female instructors, there is no evidence of an effect of the treatment on female students. Giving female students the intervention about bias had no effect on their evaluations of the female faculty. However, there is some evidence that the intervention had an effect on male students; this effect was statistically significant for both the overall rating of the instructor and the course, but not the effectiveness measure. Additionally, none of the post hoc tests of equality in the coefficients, essentially the difference-in-difference estimators, are statistically significant. In other words, the evidence does not support the conclusion that the effects of the intervention are different for male and female students when evaluating a female instructor. Of course, this may be due to the reduced power and smaller sample size for these comparisons.

**Table 3:** Treatment and gender of student effects on SET for female faculty (ordered logit models).

Variable	Instructor	Effectiveness	Course
Treatment for female students (intervention = 1)	0.44 (0.41)	0.35 (0.40)	0.48 (0.40)
Treatment for male students (intervention = 1)	1.48 (0.50)*	0.80 (0.48)	1.63 (0.49)*
Student sex (female = 1)	-0.05 (0.44)	-0.60 (0.44)	0.40 (0.44)
Threshold 1	-4.78 (1.04)	-4.13 (0.65)	-3.39 (0.63)
Threshold 2	-2.95 (0.49)	-2.97 (0.45)	-2.24 (0.42)
Threshold 3	-1.68 (0.36)	-1.86 (0.36)	-1.21 (0.34)
Threshold 4	0.67 (0.32)	0.28 (0.32)	1.13 (0.34)
N	158	158	158

* p<0.05

**Table 4:** Treatment and gender of student effects on SET for male faculty (ordered logit models).

Variable	Instructor	Effectiveness	Course
Treatment for female students (intervention = 1)	0.56 (0.59)	0.13 (0.61)	-0.03 (0.60)
Treatment for male students (intervention = 1)	-0.33 (0.57)	-0.17 (0.54)	-0.66 (0.54)
Student sex (female = 1)	-1.39 (0.55)*	-0.91 (0.55)	-1.01 (0.55)
Threshold 1	-3.51 (1.59)	-3.08 (0.53)	-5.13 (1.07)
Threshold 2	-2.04 (0.45)	-1.65 (0.40)	-3.28 (0.55)
Threshold 3	-0.58 (0.36)	0.22 (0.35)	-1.89 (0.44)
Threshold 4	-	-	-0.16 (0.38)
N	88	87	87

* p<0.05 Note that there are only three thresholds in the first two columns because no respondents used the lowest category of those questions for either of the male faculty.

The results for male instructors (Table 4) are the same as in the t-tests. There is no evidence that the treatment had any effect on the students’ evaluations of male faculty, regardless of the sex of the students. There is some evidence that female students rated the male instructors less positively than the male students did, but the treatment had no effect on this.

## Concluding remarks

The evidence from our experiment with SET suggests that a simple intervention informing students of the potential for gender biases can have significant effects on the evaluation of female instructors. These effects were consistent across two different introductory courses (one biology and one political science). These effects were substantial in magnitude; as much as half a point on a five-point scale. This effect is comparable with the effect size due to gender bias found in the literature [5]. There is no evidence of a similar effect on the evaluation of male instructors. Given the outsized role SET play in the evaluation, hiring, and promotion of faculty the possibility of mitigating this amount of possible bias in evaluations is striking.

The results about which students (self-identified male or female) are influenced by the treatment are less clear. The evidence suggests that male students are increasing their evaluation scores of female faculty, but this not a strong indicator. A larger sample size is needed to effectively test these differences. It is also possible that the students with female instructors who received the anti-bias language overcompensated their evaluations for the cues they are given. If a student is told that other students are likely to be overly negative about a female instructor, he or she may raise his evaluations to offset this potential bias.

Our expectations and the design of the intervention are driven by the assumption that the students’ biases which create gender differences in SET are implicit in nature. This is consistent with much of the existing literature to documenting gender biases in SET. The data we have, however, cannot delineate the nature of these biases. The success of the anti-bias language, which make specific allusion to the unconscious and unintentional nature of biases, may be suggestive that the students’ biases are implicit. It is also plausible that the intervention may have mitigated the use of more explicit gender biases. Regardless, the results do suggest that this intervention improved the SET scores for the female faculty.

The implication of these results is that universities should adopt some form of this language to mitigate the gender biases in SET, however, we are somewhat uncertain about the broad applicability of these results. The effects we observed may be magnified by the unusual nature of the situation for the students. It is possible that if an institution implemented widespread adoption of bias language students would be less likely to notice the language and its effects would lessen. Further research is needed to determine the most effective way to mitigate gender bias in SET on a large scale.

## Supporting information

S1 File Survey Script. (DOCX)

S1 Table Balance tests for other variables in the evaluation survey. Each entry presents the mean for the condition with the standard deviation in parenthesis. The p-value is based on a t-test where the null hypothesis is that the means are not equal.
(DOCX)
