# Supplementary material for: Mitigating gender bias in student evaluations of teaching
Source: PLoS One. 2019 May 15;14(5):e0216241. doi: 10.1371/journal.pone.0216241 (PMC6519786; doi:10.1371/journal.pone.0216241)
Supplement: S1 File — (DOCX) [file pone.0216241.s001.docx]

**S1: Survey Script**

Start of Block: Instructor

1.1 Your overall rating of this instructor is

- Very Poor (1)
- (2)
- (3)
- (4)
- Very Good (5)

1.2 What is your overall rating of the instructor's teaching effectiveness

- Almost Never Effective (1)
- (2)
- (3)
- (4)
- Almost Always Effective (5)

1.3 The instructor speaks clearly and audibly when presenting

- Almost Never (1)
- (2)
- (3)
- (4)
- Almost Always (5)

1.4 I was treated with respect in this class

- Strongly Disagree (1)
- (2)
- (3)
- (4)
- Strongly Agree (5)

End of Block: Instructor

Start of Block: Course

2.1 Your overall rating of this course is

- Very Poor (1)
- (2)
- (3)
- (4)
- Very Good (5)

2.2 How much do you feel you have learned in this course

- Almost Nothing (1)
- (2)
- (3)
- (4)
- An Exceptional Amount (5)

2.3 The textbook(s) and readings used in this course are

- Nearly Useless (1)
- (2)
- (3)
- (4)
- Extremely Useful (5)
- N/A (6)

2.4 Rate the usefulness of the **outside** assignments (homework, writings, reports and special projects, etc.) in helping you learn

- Almost Always Useless (1)
- (2)
- (3)
- (4)
- Almost Always Useful (5)
- N/A (6)

2.5 Rate the usefulness of the **in-class** activities (lectures, discussions, etc.) in this course in helping you to learn

- Almost Always Useless (1)
- (2)
- (3)
- (4)
- Almost Always Useful (5)

2.6 The difficulty level of the course is

- Among the Most Difficult (1)
- (2)
- (3)
- (4)
- Among the Easiest (5)

End of Block: Course

Start of Block: Time

3.1 On average, how many hours per week have you spent on this class, including attending classes, doing readings, reviewing notes, writing papers and any other course related work?

- Under 2 (1)
- 2-3 (2)
- 4-5 (3)
- 6-7 (4)
- 8-9 (5)
- 10-11 (6)
- 12-13 (7)
- 14-15 (8)
- 16-17 (9)
- 18 or more (10)

3.2 From the total average hours above, how many do you consider were valuable in advancing your education?

- Under 2 (1)
- 2-3 (2)
- 4-5 (3)
- 6-7 (4)
- 8-9 (5)
- 10-11 (6)
- 12-13 (7)
- 14-15 (8)
- 16-17 (9)
- 18 or more (10)

End of Block: Time

Start of Block: Student

4.1 My grade point average is:

- 1.00 - 1.75 (1)
- 1.76 - 2.25 (2)
- 2.26 - 2.75 (3)
- 2.76 - 3.25 (4)
- 3.26 - 4.00 (5)

4.2 I expect a final course grade of:

- A+ - A- (1)
- B+ - B- (2)
- C+ - C- (3)
- D (4)
- F or U (unsatisfactory) (5)
- S (satisfactory, pass) (6)

4.3 In my program this course is:

- Required (1)
- Elective (2)

4.4 My class is:

- freshman (1)
- sophomore (2)
- junior (3)
- senior (4)
- graduate student (5)
- other (6)

4.5 My major is in the area of:

- Agriculture (1)
- Business (2)
- Design (3)
- Engineering (4)
- Human Sciences (5)
- Liberal Arts and Sciences (6)
- Veterinary Medicine (7)

4.6 My sex is:

- female (1)
- male (2)

4.7 What did you like BEST about this class?

________________________________________________________________

4.8 What did you like LEAST about this class?

________________________________________________________________

End of Block: Student
