# Supplementary material for: Mitigating gender bias in student evaluations of teaching
Source: PLoS One. 2019 May 15;14(5):e0216241. doi: 10.1371/journal.pone.0216241 (PMC6519786; doi:10.1371/journal.pone.0216241)
Supplement: S1 Table — Each entry presents the mean for the condition with the standard deviation in parenthesis. The p-value is based on a t-test where the null hypothesis is that the means are not equal. (DOCX) [file pone.0216241.s002.docx]

**S1 Table**

This Table presents the balance tests for the other variables included in the SET survey. The comparisons are t-tests of the means in the anti-bias treatment and control conditions. The third column of the table presents the p-value of the t-test. None of the differences are statistically significant, suggesting that the randomization worked.

**Table S1. Balance tests for other variables in the evaluation survey**. Each entry presents the mean for condition with the standard deviation in parenthesis. The p-value is based on a t-test where the null hypothesis is that the means are not equal.

| Variable | Mean in treatment (standard deviation in parenthesis) | Mean in control (standard deviation in parenthesis) | p-value |
| --- | --- | --- | --- |
| Student sex | 0.48 (0.50) | 0.56 (0.50) | 0.24 |
| Student class | 1.33 (0.47) | -3.76 (0.60) | 0.41 |
| Expected grade | 1.85 (0.82) | 1.75 (0.85) | 0.36 |
| GPA | 4.11 (1.02) | 4.03 (1.00) | 0.52 |
| N | 128 | 118 |  |
